# A standards perspective on genomic data reusability and reproducibility

**DOI:** 10.3389/fbinf.2025.1572937

**Published:** 2025-03-10

**Authors:** Ishi Keenum, Scott A. Jackson, Emiley Eloe-Fadrosh, Lynn M. Schriml

**Affiliations:** ^1^ Department of Civil, Environmental, and Geospatial Engineering, Michigan Technological University, Houghton, MI, United States; ^2^ Complex Microbial Systems Group, National Institute of Standards and Technology, Gaithersburg, MD, United States; ^3^ Lawrence Berkeley National Laboratory, Environmental Genomics and Systems Biology Division, Berkeley, CA, United States; ^4^ Institute for Genome Sciences, University of Maryland School of Medicine, Baltimore, MD, United States

**Keywords:** reproducibility, reuse, standards, genomics, metagenomics, AI

## Abstract

Genomic and metagenomic sequence data provides an unprecedented ability to re-examine findings, offering a transformative potential for advancing research, developing computational tools, enhancing clinical applications, and fostering scientific collaboration. However, effective and ethical reuse of genomics data is hampered by numerous technical and social challenges. The International Microbiome and Multi’Omics Standards Alliance (IMMSA, https://www.microbialstandards.org/) and the Genomic Standards Consortium (GSC, https://gensc.org) hosted a 5-part seminar series “A Year of Data Reuse” in 2024 to explore challenges and opportunities of data reuse and reproducibility across disparate domains of the genomic sciences. Addressing these challenges will require a multifaceted approach, including common metadata reporting, clear communication, standardized protocols, improved data management infrastructure, ethical guidelines, and collaborative policies that prioritize transparency and accessibility. We offer strategies to enable responsible and technically feasible data reuse, recognition of data reproducibility challenges, and emphasizing the importance of cross-disciplinary efforts in the pursuit of open science and data-driven innovation.

## Introduction

We have an unparalleled opportunity through molecular techniques, specifically genome and metagenome sequencing, to identify microbial constituents in any environment. Numerous studies have highlighted the potential to leverage the vast amount of genomic and metagenomic data to discover key functions, taxa, or traits (Wu et al., 2025; Crits-Christoph et al., 2018; Altae-Tran et al., 2023). However, both technical and social issues hinder progress in the field and could impact new artificial intelligence (AI) applications. From a technical perspective, sequence data reuse is complicated by diverse data formats, inconsistencies in metadata, data quality variability, and substantial storage and computational demands. This is compounded by researchers’ attitudes and behaviors around data sharing and restricted usage, much of which disproportionately impacts early career researchers. Across social media and through journal publications, the reuse and reproducibility of genomics and metagenomics data has been a hot topic for discussion and debate (Hafner et al., 2025; Ross et al., 2024; Holden, 2024; Huttenhower et al., 2023; Amman et al., 2019). It is our opinion, that we, as data generators, have the responsibility to our future selves and to fellow researchers to generate our genomic data in a manner that can be reused. Additionally, taking into account our combined experiences and knowledge gained from these perspectives, to call out technical, social and other “gotchas” to inform researchers in the field, to provide them with a thoughtful perspective on how we, as a community, can acknowledge these challenges and outline solutions, together.

Given the numerous opportunities and challenges for data reuse, we sought to openly explore and discuss these topics with an eye towards actionable recommendations for the field. In 2024, the International Microbiome and Multi’Omics Standards Alliance (IMMSA; https://www.microbialstandards.org/) and the Genomic Standards Consortium (GSC (https://www.gensc.org/) (Schriml et al., 2020), came together to host a year-long series of community seminars and discussions entitled “A Year of Data Reuse.” The intent of the series was to explore the breadth of technical and social challenges, identifying near and long-term opportunities, and move the conversation forward on how we, as a community, can assess and improve the reuse and reproducibility of the data we generate.

## The impact of non-reusable and non-reproducible data

Scientific data reproducibility is at the cornerstone of the scientific method. Genomic sequencing, in theory, should enable an unprecedented level of reproducibility as once the data is shared publicly, scientists all around the world should be able to run the same pipelines and achieve the same result. While in many ways we have achieved this, this framework fails to account for steps in sample processing or data collection that are vital to understanding the interpretability of another’s genomic data. Future data interpretation depends on the inclusion of critical metadata when sample data is submitted to one of the International Nucleotide Sequence Database Collaboration (INSDC) resources (Karsch-Mizrachi et al., 2025). Missing, partial or incorrect metadata can lead to significant repercussions, leading to faulty conclusions about the prevalence of taxonomy or genetic inferences. Whereas, it is well documented that reporting of standardized metadata facilitates data reuse (Borry et al., 2024). Further, the laboratory methods and kits that we use to process samples can impact the resulting taxonomic community profiles (Forry et al., 2024; Forry et al., 2025; Servetas et al., 2025). Understanding the extent to which sample processing impacts the resulting genomic information can enable more nuanced interpretation of biological data.

This challenge is increasingly recognized and groups of genomics data generators in academia, government and industry have come together to discuss and solve these problems. IMMSA and GSC represent two consortia that serve to develop solutions to genomics comparability challenges. IMMSA was founded in 2016 and is an open consortium of microbiome-focused researchers from industry, academia, and government that focuses specifically on coordinating cross-cutting efforts that address microbiome measurement challenges. IMMSA members are representative experts for all major microbiological ecosystems (e.g., human/animal, built, and environmental ecosystems) and from various scientific disciplines including microbiology, bioinformatics, genomics, metagenomics, proteomics, metabolomics, transcriptomics, epidemiology, and statistics. IMMSA is made up of over 980 members and has six working groups that contribute to specific aspects of standardization from sample collection to analysis. The Genomic Standards Consortium was established, in 2005, to identify solutions to facilitate data sharing and reuse across the genomic sequence landscape, through standardized reporting of sampling and sequencing metadata. Scientists coming together organically to solve a common problem has evolved into a global community, growing to address metadata standards reporting needs as technology transformed genomic and metagenomic sequencing possibilities and scientific investigations expanded to examine soil, water, hydrocarbon, farm, food, plant and built environment microbial biodiversity, human and host associated microbial communities. The GSC defines these contextual metadata descriptions in environment and genomic specific MIxS (Minimal Information about Any (x) Sequence) standards (Yilmaz et al., 2011), that have become a unifying resource for reporting the information associated with genomics studies.

## A year of data reuse: community perspectives in 2024

The goal of the seminar series was to encourage open community discussions to identify challenges, recognize impediments to reuse and reusability and to chart out solutions. The speakers’ topics served as a framework for identifying data reuse and reproducibility challenges and solutions. Speakers were selected to present a broad array of perspectives on the topic. Each speaker was charged with identifying and speaking to their perspective as both a data generator and data reuser, on the challenges of genomic data reproducibility and reuse.

The seminar series consisted of five talks from postdoctoral researchers, staff scientists, and academic faculty, with a moderated Q&A. The seminar speakers presented their ongoing research and past studies, leaving ample time for discussion with the community. The speakers included Abraham Gihawi (University of East Anglia, United Kingdom), Robyn Wright (Dalhousie University, Canada), A. Murat Eren (Helmholtz Institute for Functional Marine Biodiversity at Oldenburg, Germany), Sushma Naithani (Oregon State University, US), and Marcus de Goffau (University of Amsterdam, Netherlands). Nearly 250 attendees participated across the five sessions, with attendance ranging from 20 to 117 (average 83) participants spanning multiple countries. The talk titles included:

• Re-Investigating Microbial Classifications in Cancer Sequencing Data
• From defaults to databases: simulated samples vs. the real world
• Reproducibility, interoperability, reusability, and flexibility in the anvi’o software ecosystem
• Omics data reuse for synthesizing plant gene-networks and pathways for the Plant Reactome Knowledgebase
• Enhancing contamination and genuine biological pattern recognition by looking elsewhere

The “Year of Data Reuse” seminar series concluded with a half day of hybrid presentations at the GSC’s annual meeting (University of Arizona, August 2024, https://genomicsstandardsconsortium.github.io/GSC24-Tucson/). The session, “Challenges of ‘Omic data reuse,” consisted of a panel discussion following presentations by three speakers: Scott Jackson (National Institute of Standards and Technology (NIST), US), Benjamin Callahan (NC State University, US), and Julie Dunning Hotopp (Institute for Genome Sciences, University of Maryland School of Medicine, US). The talk titles were:

• History of IMMSA
• Training on one study and predicting on another using publicly available microbiome datasets
• A “Research Parasite’s” Perspectives on the Challenges of Data Reuse (https://researchparasite.com)

## Data reuse and reproducibility challenges

For the purposes of these discussions, here we define “Data Reuse”: as the use of data collected by one researcher or project, being utilized by other researchers or projects, for the purpose of performing novel analysis; and define “Data Reproducibility” as the capacity and/or capability to independently run a previously published analysis, with the same samples and analysis parameters and to arrive at comparable results and conclusions.

A number of data reuse and reproducibility challenges were raised during the seminar series and through discussions at the GSC annual meeting, with the goal of informing future discussions and guiding ideas towards some initial solutions. These topics, outlined below, were identified as being critical to address these challenges, in order to move the field forward. The reusability of genomic data and the ability to reproduce the results of a study are greatly hampered when data is submitted to public archives with limited or incomplete metadata. Although the primary data is available, it is true “usability” is limited. The necessity of mining critical metadata via manual curation by either deep diving into the methods or requesting critical metadata directly from authors, was noted by a number of seminar speakers. Variability observed in laboratory, platform and kit comparative studies was highlighted as a consideration for comparing microbiome studies. Finally, in the GSC session, social challenges impacting data sharing were discussed. It was pondered how we, as a community, can incentivize colleagues to submit the breadth of metadata needed to replicate the analysis.

To begin this process, we have identified a number of data reuse challenges for researchers to keep in mind, when considering the FAIR (Findable, Accessible, Interoperable, and Reusable) data principles (Wilkinson et al., 2016).

From the reported and deposited data, is it possible to determine.

[1] Can the sequence and associated metadata be attributed to a specific sample?
[2] Where is the data and metadata found? - Supplementary files, public or private archives.
[3] Have the data access details been shared in the publication?
[4] What are the reuse restrictions (e.g., licensing) associated with the data? Can or should there be restrictions on data generated from public funding?
[5] Have the data sharing protocols and policies been defined with consistent rules that are enforced, prior to the review and journal publication?

These discussions identified a growing concern, as some genomic data are being deposited in generalist repositories, such as Zenodo (https://zenodo.org/) and OBIS (https://obis.org/), without associated, structured metadata. This practice could result in an expanding set of less usable genomic data, thus hindering data discovery and reuse. There was a special emphasis related to microbiome research, where seminar participants shared observations of variability regarding reproducing results between labs, reagent kits, analysis platforms and repeated analysis by the same researchers. It is important to consider that methods can introduce methodological variability that is greater than the natural biological variation (Forry et al., 2024; Forry et al., 2025; Rodriguez et al., 2024). Throughout the seminar discussions, we aimed to clearly outline these concerns towards integrating best practices into protocols to improve reproducible results.

Here we outline some fundamental questions for the community to consider, regarding data reproducibility:

• Can we, as a community, come to consensus on how we define the reproducibility of our data? Is this even a YES or NO answer?
• Is our data “reproducible”, if it can be shown that [1] the same or a comparable result is achieved, when the analysis is repeated, when re-using the same datasets and analysis parameters or [2] a second method finds the same result.
• Should it become “Standard SOP”, to require 2 methods, to conclude that data is reproducible.
• Should grant proposals include funding for replicate experiments.
• What is the natural biological variation of the environment, organ, or disease being reported?
• What are the key data elements needed for re-running the study? This is vital information to communicate how the data was generated. We suggest all studies should minimally report provenance (version, date, source, repository, analysis parameters) of all software components utilized in the analysis (Kanwal et al., 2017).
• Is there a publicly available Notebook or script to enable the analysis to be re-run?

## Towards best practices to address reproducibility and reuse challenges

Awareness of these challenges is the first step, to be followed by a culture shift, where we encourage reuse of our data through the inclusion of pertinent reuse information, outlined above. Framing future conversations, here we outline a number of practical ‘best practices’ solutions, to begin to outline technical solutions to address the above challenges.

1. A Genomic and Metagenomics Standards Guidebook: Looking to the future, and how we can begin to address reuse and reproducibility challenges, we are proposing that we, as a community, develop a Genomic and Metagenomic Standards Guidebook, to share best practices as a protocol for educating the upcoming generation of researchers. The guidebook could include educational modules from the National Microbiome Data Collaborative (NMDC, https://microbiomedata.org/) (Vangay et al., 2021), laboratory standards modules from NIST, genomic metadata reporting modules from the GSC, and community-specific best practices, to address the full data lifecycle from study design to data distribution.
2. Data Reuse Plan: We further propose that a Data Reuse Plan be incorporated into Data Management Plans as a part of required components for federal funding. We would encourage funding agencies to make this a required reporting component in yearly progress reports and for journals to include this information as supplementary material.
3. Reproducible Laboratory Protocols: Studies must be FAIR by design through the development of standardized protocols utilized to collect data and metadata. We identified two key areas where the reporting of key elements, namely, (i) controls and (ii) contaminants, would advance the reproducibility of future genomic and metagenomic studies. For (i) controls, inclusion of field blanks as well as positive and negative controls are critical for assessing the analytical performance of microbiome measurements. Reference materials should include “ground truth” controls (i.e., mock communities) that enable the assessment of measurement accuracy as well as complex, biomimetic, materials (e.g., feces, soil) that challenge the reproducibility of the workflow on real-word sample matrices. Additionally, it is critical to include reporting of sampling, extraction and sequencing negative controls to inform on possible sources of bias and to identify potential contamination. Reporting the type and source of microbial standards is critical for critical assessment of research findings. Three such sources include microbial measurement and microbial community standards produced by the National Institute of Standards and Technology (NIST, https://www.nist.gov/microbial-measurements), the American Type Culture Collection (ATCC, https://www.atcc.org/microbe-products/applications/microbiome-research), and Zymo Research (https://www.zymoresearch.com/collections/zymobiomics-microbial-community-standards). For (ii) contaminants, we recommend the reporting of common contaminants, for example, phiX (Bacteriophage phiX174) or common kit contaminants (e.g., the “kitome”) (Rauer et al., 2025; Duan et al., 2024).
4. Reproducible Analysis Methods: Similar to reproducible laboratory protocols, there are areas for which analyses would benefit from standardization in reporting and quantitative genomic and metagenomic comparisons. For example, an assessment of batch effects that take into account variation across sequencing runs due to technical, non-biological factors and that also affect variation in the resulting data. This type of variation occurs in batches of samples, either batches of extracted samples, or sequences. For taxonomic or functional profiling, standard reference genome and database reporting is essential, as are abundance and variability measures. Describe the differences: number of unaligned sites, # deletions, insertions, SNP variants, identical sites, % DNA-DNA hybridization. Validate the identification of strains ordered from repositories or archives utilized for analysis. Lastly, we offer the “smell test” for considering whether findings are ecologically consistent. Report the ecological and/or environmental context of the sample. Question whether the identified genomes or metagenomes are expected to be occurring in the studied environment and if the observed variability reflects the environment, host or some definitely sub-environment, specific organ or disease state. For example, in studies of the human brain consider if there is a naturally occurring or stable microbiome in this organ. Alternatively, consider if you would expect that the microbes detected in the brain are specifically related to a disease state (Lathe et al., 2023).

## Discussion

Throughout the seminar series and discussions there was a strong consensus that genomics data should align with the FAIR principles so that data can be shared. The seminar discussions fielded some initial directions for future development. It was agreed that we need to recognize and address the barriers to reuse that our community is currently supporting. A major concern, the variability and/or absence of reported metadata, was identified as a major stumbling block to data reuse. Further examination and exploration is needed to truly understand why reporting is variable, a key topic that will be advanced through both IMMSA and upcoming GSC meetings (https://www.gensc.org/pages/meetings.html).

A number of positive steps forward were identified to improve how we facilitate data reuse. These include (1) incentivising data sharing by promoting its use in publications and other scientific outputs; (2) adding new ‘data reuse’ sections to journals to highlight these efforts; (3) reporting the source (and the primary publication) when reusing data; and (4) adding a “Data Reuse Index” to CVs and to our end of year funding reports (e.g., using a BioProject citation count). One could also use tools, such as the Data Citation Explorer to identify reuse of their data found in citations incorporating uncited genomic data (Byers et al., 2024). Technically, we are suggesting a paradigm shift, for researchers to include comparability standards and relevant controls in all studies as a “best practice” in microbiome analysis. This action would provide the capacity for reproducibility assessment. As is often the case the social aspects far outweigh the technical.

Socially, genomic science is a competitive enterprise. We are incentivised to compete with our colleagues, to write the most citable paper, or seek the most research funding. As a community, through this discussion, we are striving to challenge this paradigm, to shift our perspectives as a community. We know that together we produce better, more impactful science. Together, by giving a little more, sharing a lot more, we can choose to change how we do our science. Choosing to gift our future selves with the bounty of well documented, reusable data, capturing the details that provide for the assessment of reproducibility is pivotal as we, together, explore our genomic world. It is time to ask ourselves, what we, the scientists, can do to change how we enable, report and acknowledge data reuse and how we can enhance data reproducibility by how we report our studies. This will further enable us to expand the tools at our disposal. Having well annotated and curated databases will enable us to fully leverage AI. Further, let us consider if there is a role for AI in data generation, methods extraction and in improving data reproducibility. Prior work has suggested that AI can aid in conducting metadata extraction (Islamaj et al., 2025; Gupta et al., 2024; Xiao et al., 2023), which could be leveraged as a tool for researchers conducting systematic analyses.

Lastly, let us acknowledge positive data reuse use cases. Meta-analysis, mining sequence data from publicly available resources, offers new opportunities for posing novel questions – such as, examining viral impacts on drinking water (Hegarty et al., 2022) or identifying disease-specific responses across gut microbiomes (Duvallet, 2020). Secondary analysis of datasets provides novel opportunities to discover new data associations (Skinnider, 2024) e.g,., novel host-bacteria interactions (Pilgrim, 2022) or building machine learning systems (Nieto et al., 2021).

## Data Availability

The original contributions presented in the study are included in the article/supplementary material, further inquiries can be directed to the corresponding author.
